# Usage report of pharmacopuncture in musculoskeletal patients visiting Korean medicine hospitals and clinics in Korea

**DOI:** 10.1186/s12906-016-1288-5

**Published:** 2016-08-17

**Authors:** Yoon Jae Lee, Joon-Shik Shin, Jinho Lee, Me-riong Kim, Ki Byung Park, Hwa Dong Lee, Yoonmi Lee, Jungwan Hong, In-Hyuk Ha

**Affiliations:** 1 Jaseng Spine and Joint Research Institute, Jaseng Medical Foundation, 858 Eonju-ro, Gangnam-gu, Seoul, Republic of Korea; 2 Korea Promotion Institute for Traditional Medicine Industry, Gyeongsan-si, Gyeongbuk Republic of Korea

**Keywords:** Acupuncture, Pharmacopuncture, Bee venoms, Health care costs, Complementary therapies, Korean traditional medicine

## Abstract

**Background:**

Pharmacopuncture is a relatively new acupuncture therapy combining acupuncture with herbal medicine. While pharmacopuncture is applied extensively in Korean medicine treatment, there are no clinical reports regarding what types of pharmacopuncture are used for which diseases.

**Methods:**

Data was extracted retrospectively from the electronic medical records of all inpatients and outpatients at 12 Korean medicine hospitals and clinics during the period of December 17, 2010 to October 2, 2014. Treatment patterns for acupuncture, electroacupuncture and pharmacopuncture were analyzed. Principle diagnosis codes, frequency of treatment, pharmacopuncture type and costs were investigated to assess pharmacopuncture use in clinical settings.

**Results:**

During the study period, a total 33,415 inpatients and 373,755 outpatients visited the study sites, and most were musculoskeletal. Among inpatients and outpatients, 98.6 % and 77.6 % received pharmacopuncture, respectively. Administration rate of pharmacopuncture for the 10 most frequent principle diagnosis codes was 97.2–99.3 % in inpatients, and that for outpatients was 73.0–91.5 %. The average number of pharmacopuncture sessions in pharmacopuncture recipients was 8.2 ± 12.3 for outpatients and 25.8 ± 18.7 for inpatients. The mean total cost for pharmacopuncture per patient was $556.24 ± 174.62 among inpatients, and $149.16 ± 243.85 among outpatients. Estimated average cost per pharmacopuncture session was $23–24 for inpatients, and $17–18 for outpatients. Shinbaro1, bee venom, Hwangryunhaedok, and Shinbaro2 were the most frequently used pharmacopuncture types.

**Conclusions:**

This is the first analysis of treatment patterns of pharmacopuncture in a large-scale Korean medicine hospital/clinic patient population. We verified patterns of pharmacopuncture use for musculoskeletal disease treatment in Korea, and use of pharmacopuncture varied depending on disease or symptom severity. These results are expected to contribute to future clinical study design and standardization of pharmacopuncture.

## Background

Acupuncture is a treatment procedure where needles are inserted at acupoints through the skin, and has been used mainly in and around East Asia with a history of at least 2000 years. Based on its long history and widespread use of CAM throughout the world, various new acupuncture techniques are being developed. One such relatively new acupuncture technique is pharmacopuncture (or herbal acupuncture) which combines acupuncture and herbal medicine by injecting herbal extracts into acupoints. Early forms of pharmacopuncture started in China in the early 1950s and were referred to as acupoint injection or aqua-puncture [1, 2]. Pharmacopuncture was later introduced to Korea and adopted by Korean medicine doctors through Nam in the 1960s [3]. Use is considered to have become widespread since the 1990s, and the fast-paced growth of pharmacopuncture is generally attributed to clinical observation of its effectiveness and experimental evidence of efficacy for various conditions [4]. While pharmacopuncture has extended use to various diseases in Korea, there are no clinical reports regarding how much of which types of pharmacopuncture are used at what cost, or for which diseases. Official insurance claims statistics for pharmacopuncture are only available for automobile insurance coverage, and it was reported that approximately 1,555,000 pharmacopuncture sessions were administered to 168,089 patients for related treatments in 2014 [5]. Though these numbers suggest that pharmacopuncture is applied extensively in clinical practice, it is difficult to establish specific indications for pharmacopuncture use as costs are mainly paid out-of-pocket. The major applications of pharmacopuncture can be inferred from previous studies, and Kim et al. [4] reported in a descriptive analysis of pharmacopuncture studies in Korea that it was used for a wide range of diseases, from lumbar, immune system and gynecological disorders to cancer-related diseases. Also, a 2011 meta-analysis was conducted on the effect of pharmacopuncture in asthma [6]. While a study of pharmacopuncture-related RCTs published in relevant academic journals in Korea similarly reported that pharmacopuncture was applied to various diseases including functional headache and dysmenorrhea, the majority of clinical studies concerned musculoskeletal disorders [7]. This is consistent with reports that Korean medicine (KM) is used most commonly for musculoskeletal disorders in Korea [8], and pharmacopuncture is also considered to be used mainly for musculoskeletal disorders in light of previous reports on the effect of pharmacopuncture for such disorders as lumbar intervertebral disc displacement (IDD) [9], degenerative knee osteoarthritis [10], and ankle sprain [11]. Moreover, according to a recent survey on practice patterns of IDD treatment, 95.9 % of responding Korean medicine doctors (KMDs) replied that they incorporated pharmacopuncture in their practice for lumbar IDD, suggesting that pharmacopuncture is commonly used for IDD [12]. A clinical guideline developed in Korea also recommends bee venom, Soyeom (消炎), Joongseongouhyul (中性瘀血), and Shinbaro pharmacopuncture for IDD at recommendation level B [13].

Although it can be surmised that pharmacopuncture is a regular part of KM practice, currently reports on clinical practice patterns are scarce. Although the abovementioned survey on IDD was an investigation on pharmacopuncture use [12], usage amounts were estimates based on KMD opinion and not supported by concrete data sources such as medical records. Due to paucity of investigations on pharmacopuncture type, application, duration, and dosage, there is increased difficulty in standardization of pharmacopuncture administration method and development of clinical practice guidelines. More consideration should be given to wider insurance coverage of pharmacopuncture to reduce financial burden, but lack of data on clinical application is an obstacle to health insurance inclusion, potentially leading to increase in health-related costs. KM has yet to establish standardized pharmacopuncture guidelines by disease, and this study therefore attempted to comprehensively evaluate clinical trends of pharmacopuncture use in musculoskeletal disorders. The study outcomes are expected to contribute as basic information to determining health insurance coverage for pharmacopuncture and standardization of pharmacopuncture.

## Methods

Data was extracted retrospectively from all inpatient and outpatient electronic medical records (EMRs) at 12 sites of KM hospitals and clinics from December 17, 2010 to October 2, 2014 including basic patient characteristics (sex, age), outpatient visit information (treatment duration, number of visits, principle diagnosis code, and treatment contents regarding acupuncture, electroacupuncture, and pharmacopuncture), and hospitalization information (duration of hospital stay, number of admissions, and treatment contents regarding acupuncture, electroacupuncture, and pharmacopuncture). Study sites were sampled from 12 KM hospitals and clinics covering 5 districts including Seoul (Gangnam, Nowon, Mok-dong, Yeongdeungpo and Jamsil), Daejeon, Busan (Haeundae), Changwon and Gyeonggi (Bucheon, Bundang, Suwon and Ansan), in order to gain insight to nationwide trends by securing a maximum number of sites. Considering for potential difference in prescription trends by visit type, subjects were analyzed as inpatients and outpatients, and principle diagnosis codes, number of treatment sessions, and costs were investigated to assess use of pharmacopuncture in clinical settings. Disease information conformed to EMR principle diagnosis codes following the Korean Standard Classification of Diseases (KCD), which corresponds to the 10th revision of the International Standard Classification of Diseases (ICD-10), and in absence of principle diagnosis code, diagnosis code of highest frequency served as the principle diagnosis code in analysis. Number of outpatient visits was tallied as number of visits during the treatment period, hospital stay duration was defined as total number of days from date of admission to discharge, admission number as total number of admissions, and sessions of acupuncture and pharmacopuncture was totaled as the number of prescriptions during hospital visits or stay. Many different prescription codes refer to pharmacopuncture treatment procedures, and for this reason KMDs in clinical practice classified prescription code names by pharmacopuncture type prior to analysis. As multiple pharmacopuncture types may be used in an individual patient for different symptoms, pharmacopuncture frequency was analyzed from total pharmacopuncture treatment prescriptions per patient. Some patients were included in both the inpatient and outpatient groups as it was common for discharged inpatients to continue treatment as outpatients. The authors attempted to reduce bias by mutually exclusively analyzing all acupuncture and pharmacopuncture prescriptions during hospital stay as inpatient cases, and all during outpatient visits as outpatient cases. Included participants were covered by a variety of insurances including national health insurance, medicaid, and automobile insurance, and all patient data was included for analysis regardless of insurance type. In cost analysis, however, while acupuncture and electroacupuncture have fixed costs as reimbursement items in Korea, pharmacopuncture costs are either paid entirely out of pocket or covered by automobile insurance. For this reason, pharmacopuncture treatment codes for automobile insurance coverage were excluded from cost analysis. All costs are presented as U.S. dollars at the exchange rate as of December 2015 (1175.50 Korean won = 1 U.S. dollar).

## Results

### Participants

A total 33,415 inpatients and 373,755 outpatients were included as study subjects from December 17, 2010 to October 2, 2014. Among inpatients, 15,043 (45 %) were male and 18,372 (55 %) female, and among outpatients, 47.7 % were male and 52.3 % female. Average age of inpatients was 45.6 ± 14.0 years, slightly older than the 43.9 ± 15.4 years of the outpatient group. Average length of hospital stay was 19.0 ± 12.5 days, and the average number of admissions in inpatients was 1.3 ± 0.8 times. In outpatients, the average number of visits was 7.5 ± 11.9 times (Table 1).

**Table 1:** Patient characteristics and number and duration of treatment

	Inpatient (*n* = 33,415)	Outpatient (*n* = 373,755)
Male (*n*, (%))	15,043 (45)	178,384 (47.7)
Female (*n*, (%))	18,372 (55)	195,371 (52.3)
Age (mean ± SD, years)	45.6 ± 14.0	43.9 ± 15.4
Number of treatments	1.3 ± 0.8	7.5 ± 11.9
Treatment duration (mean ± SD, days)^a^	19.0 ± 12.5	

^a^Treatment duration in the ambulatory department is not shown as the calculation method is likely to overestimate duration

### Frequency of acupuncture, electroacupuncture, and pharmacopuncture treatment

Of 33,415 inpatients, 32,947 patients received 1 or more sessions of pharmacopuncture, which correlated to 98.6 % of total inpatients. A total 99.4 % of inpatients were administered acupuncture, 85.9 % electroacupuncture, and 98.6 % pharmacopuncture, showing that almost all inpatients received both acupuncture and pharmacopuncture. Average number of pharmacopuncture treatment sessions during hospital stay was 22.8 ± 18.4 times, and the median was 19 times, indicating that though most patients underwent pharmacopuncture treatment once a day, some received pharmacopuncture twice or more a day considering that average hospital stay was 19.0 ± 12.5 days.

Among 373,755 outpatients, 85.3 % received acupuncture, 72.8 % electroacupuncture, and 77.6 % pharmacopuncture. In recipients of pharmacopuncture, the average number of pharmacopuncture treatment sessions was 8.2 ± 12.3 times (median 3). While frequency of acupuncture was comparable to that of pharmacopuncture in the inpatient group, it was higher than that of pharmacopuncture in outpatients (Table 2).

**Table 2:** Frequency of acupuncture, electroacupuncture, and pharmacopuncture

		Inpatient (*n* = 33,415)	Outpatient (*n* = 373,755)
Acupuncture	*n* (%)	33,198 (99.4 %)	318,764 (85.3 %)
	sessions (mean ± SD)	25.8 ± 18.7	8.0 ± 12.2
	median (quantile)	22 (11, 37)	3 (1, 10)
Electroacupuncture	*n* (%)	28,703 (85.9 %)	272,033 (72.8 %)
	sessions (mean ± SD)	15.4 ± 13.0	7.9 ± 11.9
	median (quantile)	12 (6, 21)	3 (1, 10)
Pharmacopuncture	*n* (%)	32,947 (98.6 %)	289,860 (77.6 %)
	sessions (mean ± SD)	22.8 ± 18.4	8.2 ± 12.3
	median (quantile)	19 (9, 31)	3 (1, 10)

### Number of pharmacopuncture sessions by major disease and principle diagnosis code

The 10 most frequent principle diagnosis codes among inpatients were M51, S33, M54, M50, S13, M48, M17, M47, S83, and M25, in order of frequency, and a total 88.5 % of inpatients fell under these principle diagnosis code categories. The 10 most frequent principle diagnosis codes in outpatients were S33, M51, M54, S13, M50, S93, S83, M25, S43, and M48, in order of frequency, diverging from the order presented in the inpatient group. In outpatients, principle diagnosis codes involving joint disorders such as shoulder and ankle joint were more common. In addition, 81.4 % of outpatients were covered by these 10 principle diagnosis codes, suggesting that the ambulatory department displayed wider range in diagnosis code. The proportion of patients receiving acupuncture and pharmacopuncture, respectively, were highly similar for each principle diagnosis code in inpatients, and proportion for pharmacopuncture was slightly lower for S13 and S83 compared to other diagnosis codes. However, the proportion of patients receiving acupuncture was over 10%p different from that of pharmacopuncture for S33, S13, S93, S83, and S43, compared to small difference in M51, suggesting that pharmacopuncture use is determined by severity or duration of disease (Table 3).

**Table 3:** Acupuncture and pharmacopuncture usage by principle diagnosis code

Principle diagnosis code	Number of patients	Acupuncture (*n* (%))	Pharmacopuncture (*n* (%))	Principle diagnosis code	Number of patients	Acupuncture (*n* (%))	Pharmacopucture (*n* (%))
Inpatients				Outpatients			
Total	33,415	33,198	32,947	Total	373,755	318,764	289,860
M51	12,597 (37.7)	12,547 (99.6)	12,495 (99.2)	S33	94,536 (25.3)	88,234 (93.3)	78,639 (83.2)
S33	7774 (23.3)	7726 (99.4)	7662 (98.6)	M51	61,844 (16.5)	57,275 (92.6)	56,062 (90.7)
M54	2708 (8.1)	2684 (99.1)	2657 (98.1)	M54	61,213 (16.4)	53,203 (86.9)	49,371 (80.7)
M50	2475 (7.4)	2461, (99.4)	2451 (99.0)	S13	31,057 (8.3)	31,057 (93.5)	25,530 (82.2)
S13	1307 (3.9)	1297 (99.2)	1271 (97.2)	M50	18,589 (5.0)	18,589 (93.5)	17,007 (91.5)
M48	695 (2.1)	692 (99.6)	690 (99.3)	S93	9159 (2.5)	9159 (93.0)	6682 (73.0)
M17	561 (1.7)	557 (99.3)	553 (98.6)	S83	7762 (2.1)	7762 (90.9)	4074 (80.7)
M47	539 (1.6)	536 (99.4)	531 (98.5)	M25	7182 (1.9)	7182 (89.6)	3065 (81.4)
S83	461 (1.4)	455 (98.7)	450 (97.6)	S43	6710 (1.8)	6710 (92.1)	6263 (82.2)
M25	433 (1.3)	431 (99.5)	430 (99.3)	M48	5871 (1.6)	5871 (91.5)	5843 (88.2)

M51: Other intervertebral disc disorders, S33: Dislocation, sprain and strain of joints and ligaments of lumbar spine and pelvis, M54: Dorsalgia, S13: Dislocation, sprain and strain of joints and ligaments at neck level, M48: Other spondylopathies, M17: Gonarthorosis [arthrosis of knee], M47: Spondylosis, S83: Dislocation, sprain and strain of joints and ligaments of knee, M25: Other joint disorders, NEC, S93: Dislocation, sprain and strain of joints and ligaments at ankle and foot level, S43: Dislocation, sprain and strain of joints and ligaments of shoulder girdle

### Types of pharmacopuncture

The most commonly used types of pharmacopuncture among inpatients were Shinbaro1, Hwangryunhaedok, bee venom, Shinbaro2, and Joongseongouhyul in order of frequency, and in outpatients, Shinbaro1, bee venom, Hwangryunhaedok, Shinbaro2, and Joongseongouhyul were used most frequently in this order (Table 4). The preparation method for each pharmacopuncture type is shown in Table 5. All pharmacopuncture solutions were prepared in cleanrooms at Jaseng herbal dispensary, an extramural facility adhering to Korean Good Manufacturing Practice (K-GMP) standards.

**Table 4:** Most frequently used pharmacopuncture types

Pharmacopuncture in inpatient care *n* (%)		Pharmacopuncture in outpatient care *n* (%)	
Total	32,947 (100)	Total	289,860 (100)
Shinbaro 1	17,733 (53.8)	Shinbaro 1	80,893 (27.9)
Hwangryunhaedok (黃蓮解毒)	11,596 (35.2)	Bee venom	49,279 (17.0)
Bee venom	10,560 (32.1)	Hwangryunhaedok (黃蓮解毒)	42,173 (14.5)
Shinbaro2	4622 (14.0)	Shinbaro2	25,188 (8.7)
Joongseongouhyul (中性瘀血)	2001 (6.1)	Joongseongouhyul (中性瘀血)	19,418 (6.7)
Hominis placenta (紫河車)	1233 (3.7)	Hominis placenta (紫河車)	8788 (3.0)
Shinbaro 3	1137 (3.5)	Muscle relaxation	4585 (1.6)
Muscle relaxation	358 (1.1)	Shinbaro3	4550 (1.6)

**Table 5:** Preparation of frequently used pharmacopuncture solutions

	Preparation methods
Shinbaro 1	Herbal extraction (Cibotium barometz, Saposhnikovia divaricata, Eucommia ulmoides, Acanthopanax sessiliflorus, Ostericum koreanum, Angelica pubescens, Achyranthes japonica, Paeonia lactiflora) using reflux extraction with 70 % spiritus vinosus → progressive purification using alcohol immersion → freeze-drying → dilution → filtering → sterilization
Shinbaro 2	Herbal extraction (Cibotium barometz, Saposhnikovia divaricata, Eucommia ulmoides, Acanthopanax sessiliflorus, Ostericum koreanum, Angelica pubescens, Achyranthes japonica, Paeonia albiflora, Scolopendra subspinipes) using reflux extraction with 70 % spiritus vinosus → progressive purification using alcohol immersion → freeze-drying → dilution → filtering → sterilization
Shinbaro 3	Herbal extraction (Harpagophytum procumbens) extraction using reflux extraction with 70 % spiritus vinosus → progressive purification using alcohol immersion → freeze-drying → dilution → filtering → sterilization
Bee venom	Purification of dried bee venom → freeze-drying → dilution → filtering
Hwangryunhaedok (黃蓮解毒)	Herbal distillation extraction (Coptis japonica, Scutellaria baicalensis, Phellodendron amurense, Gardenia jasminoides) → filtering → sterilization
Joongseongouhyul (中性瘀血)	Herbal distillation extraction (Gardenia jasminoides, Commiphora molmol, Boswellia carterii, Corydalis ternate, Paeonia obovata, Salvia miltiorrhiza, Caesalpina sappan) → filtering → sterilization
Hominis placenta (紫河車)	Hominis placenta extracts approved for herbal medicine preparation → filtering → sterilization
Muscle relaxation	Herbal extraction (Paeonia lactiflora, Glycyrrhiza uralensis) using reflux extraction with 70 % spiritus vinosus → progressive purification using alcohol immersion → freeze-drying → dilution → filtering → sterilization

### Pharmacopuncture-related costs

As pharmacopuncture cost differs by insurance, this study chose to exclude patients receiving pharmacopuncture treatment under automobile insurance coverage, and accordingly analyzed pharmacopuncture costs in 32,246 inpatients and 229,219 outpatients. Pharmacopuncture costs for the entire duration of treatment for each patient were totaled, and the average total cost filed in insurance claims was $556.24 ± 174.62 per patient in inpatients, and $149.16 ± 243.85 in outpatients.

## Discussion

Pharmacopuncture is a relatively new acupuncture format that embodies aspects of both manual acupuncture and herbal medicine by injecting purified herbal extracts according to herbal medicine properties and flavors at acupoints relevant to the disease. Its efficacy is considered to take synergistic effect by implementing and combining the physical effect of acupoint stimulation and the chemical effect of herbal extracts. There are reports that pharmacopuncture heightens the clinical effect of acupuncture when combined with acupuncture compared to acupuncture alone, and pharmacopuncture is drawing attention as a possible solution for various refractory diseases [14]. While it can be conjectured that pharmacopuncture usage is high for various diseases from an overview of pharmacopuncture studies conducted in Korea, there are no reports providing clinical information as to which types of pharmacopuncture are used at which acupoints for what diseases. Analysis of previous clinical studies on pharmacopuncture shows that per acupoint and total pharmacopuncture volume are highly variable from 0.01 to 0.5 cc with one study reporting injection of 3.5 cc intra-articularly, and from 0.01 to 1.5 cc, respectively [15]. Meanwhile, a recent survey on pharmacopuncture use for IDD in KMDs reported ranges between 1.3 and 3.5 cc, likewise displaying a wide range [12]. While these figures may be a reflection of differences in disease, patient symptoms or severity, it may also be taken as an indication of absence of a standardized procedure. Moreover, previous studies are highly heterogeneous with regard to intervention with pharmacopuncture agents ranging from single solution ingredients such as hominis placenta and bee venom, to herbal mixtures such as Hwangryunhaedok and Joongseongouhyul, and preparation methods were likewise diverse [15]. Taking these circumstances into account, standardization of pharmacopuncture solution preparation methods and establishment of standard pharmacopuncture procedures by disease would seem to deserve high priority, and efforts are steadily being put toward standardization and improvement of pharmacopuncture efficacy and stability [16–19]. The multiform methods of administration are an added difficulty to standardization and potential inclusion of pharmacopuncture in national insurance coverage. While standardization of administration procedure by disease in addition to solution preparation is a pressing matter, the exact amount of pharmacopuncture used in clinical settings is yet unclear due to noncoverage. The study sites included KM hospitals specializing in spine disorders designated as such by the Korean Ministry of Health and Welfare, and all study sites were KM hospitals and clinics that treated a large number of musculoskeletal patients on a regular basis. A 2009 review of Korean RCTs on pharmacopuncture showed that studies on patients with musculoskeletal disorders such as degenerative knee osteoarthritis, low back and/or leg pain, shoulder and/or arm pain, and ankle sprain were most frequent [7], and taking into account that Korean medicine is mainly used for musculoskeletal disorders in Korea, these KM hospitals were considered suitable sites for investigation of current pharmacopuncture usage. This study investigated current pharmacopuncture use by assessing the electronic medical records of all patients visiting 12 KM hospitals and clinics situated throughout the country from December 17, 2010 to October 2, 2014.

Of included participants, 33,415 were inpatients and 373,755 outpatients, of whom 98.6 and 77.6 % received pharmacopuncture, respectively. The average number of pharmacopuncture sessions was 22.8 ± 18.4 times (median: 19) among inpatients, and 8.2 ± 12.3 times (median: 3) among outpatients. The rate of pharmacopuncture administration by principle diagnosis codes was highest among inpatients in M51, S33, M54, S13, and M48, in this order, and rates varied between 97.2 and 99.3 % for the 10 most frequent principle diagnosis codes, showing small variance. Among outpatients, the rate was highest in order of S33, M51, M54, S13, M50, S93, M17, M47, S83, and M25, and variance was larger at 73.0 to 91.5 % than that of inpatients in rate of pharmacopuncture by 10 most frequent principle diagnosis codes. The reason for this significant divergence in pharmacopuncture usage rate between inpatients and outpatients was inferred to be due to difference in severity of disease. For instance, the rate of pharmacopuncture for minor diseases such as sprain was lower than that of acupuncture in outpatients which suggests that severity of disease is an important factor in determining use of pharmacopuncture, i.e. that pharmacopuncture is generally used more frequently in patients with severe pain to the aim of resolving such issues. Treatment duration for the ambulatory department was not analyzed as treatment period is diverse by patient characteristics and disease severity, and relapse or long-term management is common in musculoskeletal disease with patients frequently seeking medical attention up to several months after initial onset, and resulting in overestimation of period. Meanwhile, frequency of pharmacopuncture use was higher than that of acupuncture in outpatients, possibly as patients receiving pharmacopuncture suffered more severe symptoms requiring more treatment to achieve clinical effect. In addition, considering that the proportion of patients receiving pharmacopuncture (77.6 %), which is paid entirely out of pocket and consequently has lower accessibility compared to acupuncture or electroacupuncture which are covered by national health insurance, was higher than that of electroacupuncture (72.8 %), which incurs only $1–2 in self-payment cost, out of total patients, it may be carefully inferred that KMDs in Korea regard pharmacopuncture to be as or more clinically effective than electroacupuncture.

In analysis of pharmacopuncture-related costs in 32,246 inpatients and 229,219 outpatients, average total pharmacopuncture cost billed per patient during the treatment period was about $556.24 ± 174.62 among inpatients, and $149.16 ± 243.85 among outpatients. While direct comparison between inpatient and outpatient costs is not possible as those covered by automobile insurance were excluded from cost analysis and number of subjects therefore differs, but considering the average frequency of treatment in the inpatient/outpatient department, the estimated average cost per pharmacopuncture session was $23–24 in inpatients, and $17–18 in outpatients. As these hospitals leaves decisions on pharmacopuncture cost pending on overall pharmacopuncture volume and number of acupoints to physicians within a preset range, it is suggested that cost in inpatients was generally higher as they presented more severe cases.

In analysis of pharmacopuncture type, Shinbaro1, bee venom, Hwangryunhaedok, and Shinbaro2 were shown to be most frequently used. Shinbaro1 and 2 solutions contain the herbal compound GCSB-5, derived from Chungpa-jun. The effects of GCSB-5 in anti-inflammation, neuroprotection, and articular cartilage protection have been reported in various experimental studies [20–22], and Shinbaro pharmacopuncture has similarly been proven to be effective for IDD [14, 23] and arthritis [24] through clinical research which explains the frequent use in M51 and M17 patients. The safety of Shinbaro pharmacopuncture has been demonstrated in single intramuscular dose toxicity tests [25] and repeated intramuscular dose toxicity tests [26]. The effects of bee venom [27] and Hwangryunhaedok [28] in spinal diseases have also been reported, indicating that clinically preferred pharmacopuncture types are mainly evidence-based. It may be further conjectured that use of evidence-based pharmacopuncture is gaining in popularity amongst KMDs through heightened credibility drawing from various clinical study results [4]. It has also been suggested that pharmacopuncture is used primarily for musculoskeletal disorders given the fact that the principle diagnosis codes in pharmacopuncture treatment were musculoskeletal. In addition, this study found that commonly used pharmacopuncture solutions were prepared through reflux extraction with 70 % spiritus vinosus, distillation extraction, or isolation and purification. While such methods as low-temperature, and pressure extraction are also used for pharmacopuncture solution preparation in Korea [29], only use of reflux extraction with 70 % spiritus vinosus, distillation extraction, or isolation and purification was confirmed in this study as the included KM hospitals and clinics did not use low-temperature extraction and pressure extraction.

However, this study holds the following limitations. Only principle diagnosis codes were included in analyses and though patients were each assigned one principle diagnosis code during the study period of 2010 to 2014, the disease is likely to have progressed or changed over time in some considering the length of the data extraction period, and the computerized analysis used in this study was not equipped to capture such changes. There is the further limitation that changes in pharmacopuncture type reflecting physician judgment on disease and symptoms could be not detected, and as a result, the pharmacopuncture types most commonly used for each principle diagnosis code could not be established. Moreover, while it was deduced that disease severity may play an influential role in deciding pharmacopuncture use, the possibility that some patients may have refused pharmacopuncture for economic reasons due to noncoverage cannot be excluded. In addition, this study was unable to collect data on the volume of pharmacopuncture solution used for treatment or the number of acupoints to which the treatment was applied. Still, results of a survey on KMDs employed at the current study sites that 2.9–5.8 acupoints were chosen for pharmacopuncture in IDD patients with 1.2–3.9 cc of pharmacopuncture solution injected at each session may be of reference [12].

Many of the hospitals included in this study were spine specialty KM hospitals, and the hospitals accordingly treated a large number of IDD (especially M51) and spinal stenosis patients, which may partially explain the markedly high frequency of Shinbaro pharmacopuncture use. Although this study covered a large number of KM hospitals and patients, most focused on spinal disorders and trends may be disparate from general KM settings. Nevertheless, KM is mainly used for musculoskeletal disorders in Korea, and this study is the first large-scale study on pharmacopuncture use in electronic medical data from KM hospitals specializing in musculoskeletal disease treatment and holds significance as the first report on use of pharmacopuncture for musculoskeletal disorders. The results of this study are expected to contribute to standardization of treatment procedures involving pharmacopuncture, future clinical research design, and inclusion of pharmacopuncture in Korean national health insurance coverage.

## Conclusions

This is the first large-scale analysis on treatment patterns of pharmacopuncture in KM hospital/clinic patients. We determined practice patterns regarding pharmacopuncture used for musculoskeletal disease treatment in Korea, and trends of pharmacopuncture use were shown to depend on severity of disease or symptoms. These results are expected to contribute to future clinical study design and standardization of pharmacopuncture preparation and practice.

## Abbreviations

EMR: Electronic medical record
ICD: International Standard Classification of Diseases
IDD: Intervertebral disc displacement
KCD: Korean Standard Classification of Diseases
K-GMP: Korean Good Manufacturing Practice
KMD: Korean medicine doctor
